# Genome-wide development of SSR molecular markers for modern sugarcane cultivars

**DOI:** 10.3389/fpls.2025.1573967

**Published:** 2025-04-01

**Authors:** Yi Xu, Siyuan Chen, Shuqi Chen, Xiangzhen Wei, Heyang Shang, Qing Zhang, Jisen Zhang

**Affiliations:** ^1^ State Key Laboratory for Conservation and Utilization of Subtropical Agro - bioresources, Guangxi University, Nanning, China; ^2^ College of Life Science and Technology, Guangxi University, Nanning, China; ^3^ College of Agriculture, Guangxi University, Nanning, China

**Keywords:** modern sugarcane cultivars, genome-wide, SSR markers, polymorphism, molecular breeding

## Abstract

Modern sugarcane cultivars are derived from interspecific hybridization between *S. officinarum* and *S.* sp*ontaneum* with complex genetic backgrounds, and their lack of SSR markers limits the genetic improvement of sugarcane. In this study, We searched for and identified SSR loci within the genomes of 14 Poaceae plants. Notably, a significant positive correlation (r = 0.958) was detected between genome size and the number of SSRs. We identified SSR loci in the whole genome of XTT22, a modern sugarcane cultivar. A total of 1,054,918 SSR loci were identified, with a frequency of 123 loci/Mb and an average of 1 SSR locus per 8.11 kb, with Chr1 having the highest content and frequency of SSR loci. Among different repeat types, the number of mononucleotide repeats (620, 901) and dinucleotide repeats (238, 261) was the largest, accounting for 81.45% of the total number of SSR loci, and the number of SSR decreases with the increase of the number of SSR repeat motifs. Based on the above SSR loci, 910,519 primer pairs were obtained, and 459 SSR markers with polymorphism were screened. The polymorphism rate of SSR markers among different SSR repeat types ranged from 81.97% to 97.90%, and the pentanucleotide repeat type had the highest number of SSR markers. In order to test the universality of the developed SSR markers in sugarcane and its related species, 24 polymorphic SSR markers were randomly selected for verification in 33 sugarcane and its related species and amplified 134 alleles in total. Each pair of primers amplified 1-11 alleles, with an average of 5.58 alleles per pair. This study is the first to systematically develop SSR molecular markers for modern sugarcane cultivars at the genome-wide level, which not only enriches the number of existing SSR markers of modern sugarcane cultivars, but also provides important molecular markers to support the molecular marker-assisted breeding of sugarcane.

## 1 Introduction

Sugarcane is the most important sugar crop in China. As the world’s third-largest producer of sugar, China derives 90% of its sugar from sugarcane (Hu, 2018). Sugarcane is also a highly promising energy crop and one of the most efficient crops in converting solar energy into usable chemical energy. It accounts for over 40% of the world’s total biomass ethanol production and is a vital component of the biomass ethanol industry. Compared to other energy crops, sugarcane demonstrates outstanding advantages in ethanol yield and production costs, making it widely recognized as one of the most excellent renewable bioenergy crops worldwide (Zhang et al., 2018b).

SSR markers are DNA fragments composed of repetitive nucleotide motifs ranging from 1 to 6 base pairs, and they are present in the genomes of prokaryotes and eukaryotes (Xu et al., 2021b). SSR markers have advantages such as high polymorphism, high specificity, simplicity of operation, relatively low cost, easy detection, and genetic co-dominance. They have been widely used in population differentiation, genetic diversity studies, kinship analysis, linkage analysis, gene mapping, and genetic diversity research, providing convenience for molecular breeding of various crops (Xu et al., 2021b). SSR molecular markers have been used in various plants such as wheat (*Triticum aestivum*) (Xu et al., 2021a), barley (*Hordeum vulgare*) (Mohammadi et al., 2020), rice (Oryza sativa) (Li et al., 2023), cotton (*Gossypium* spp.) (Wang et al., 2023a), and maize (*Zea mays*) (Belalia et al., 2018) to construct genetic linkage maps, perform variety identification, and analyze genetic diversity. In wheat hybrid populations, SSR markers were used for QTL identification, and six QTLs were identified for leaf rust resistance, which can be directly applied in marker-assisted selection (MAS) for resistance studies (Mohammadi et al., 2020). In peanut, genetic diversity analysis of 101 varieties was conducted using 40 SSR markers, and an evolutionary tree was constructed based on genotyping data, providing a basis for assessing the genetic relationships among varieties and selecting breeding materials (Hong et al., 2021).

Currently, most of the developed SSR markers in sugarcane are EST-SSR markers (Jiang et al., 2023), as well as SSR markers developed based on haploid genomes or closely related species. Previous studies have identified numerous SSRs in primitive sugarcane varieties (Liu et al., 2022). However, these SSRs have not been effectively applied in the molecular breeding of sugarcane cultivars. Therefore, the development of SSR markers based on the whole genome of sugarcane is still in its early stages and is not yet mature. These markers have low polymorphism, low repeatability, and lack richness in the genome. The modern sugarcane cultivar XTT22 has the longest cultivation history and is a key variety in most modern sugarcane cultivars due to its excellent economic traits. Therefore, in this study, SSR markers based on the XTT22 genome were developed with the aim of enriching the existing repertoire of SSR markers in the sugarcane genome. These markers serve as important resources for the construction of sugarcane genetic maps and the screening of molecular markers for marker-assisted breeding.

## 2 Materials and methods

### 2.1 Data sources

In this study, we utilized a variety of materials including 14 Poaceae plants, comprising sugarcane varieties, sugarcane related species, and representative Poaceae species. The sugarcane varieties consisted of one modern sugarcane cultivar and two each of *S. officinarum* and *S.* sp*ontaneum*. The sugarcane related species included *Erianthus rufipilus* and *Saccharum arundinaceum.* Representative Poaceae species encompassed barley (*Hordeum vulgare*), *Aegilops tauschii*, maize (*Zea mays*), *Sorghum bicolor*, millet (*Setaria italica*), rice (*Oryza sativa*), and *Brachypodium distachyon*.

The genome data for *S.* sp*ontaneum* (AP85-441, Np-X), *S. officinarum* (LA-purple, Badila), *Hordeum vulgare*, *Aegilops tauschii*, *Zea mays*, *Sorghum bicolor*, *Setaria italica*, *Oryza sativa*, *Brachypodium distachyon* and *E. rufipilus* (YN2009-3) were obtained from the NCBI database (https://www.ncbi.nlm.nih.gov/). The genomic data for modern sugarcane cultivation varieties (XTT22) can be obtained at sugarcane.gxu.edu.cn/scdb/, and *S. arundinaceum* (HN92-105) have not been published.

### 2.2 SSR loci identification and primer design

This study employed the MISA (Microsatellite identification tool) software (http://pgrc.ipk-gatersleben.de/misa) to scan SSR (Simple Sequence Repeat) loci in the whole genome sequences of 14 Poaceae species. The least numbers of SSRs used for identification of mono-, di-, tri-, tetra-, penta-, hexanucleotides and compound type were 10, 7, 6, 5, 4, 4 and 2, respectively, with a maximum spacing of 100 bp between adjacent SSRs. Next, using the identified flanking conserved sequences of the SSR sites from the entire genome of the XTT22, we utilized the Primer3.0 software (http://primer3.sourceforge.net/releases.php) for batch development of SSR primers. The primer design parameters were set as follows: the desired product length ranged from 100 to 300 bp, the primer length ranged from 18 to 23 bp, and the GC-content was set between 40% and 60%. The optimal annealing temperature was set at 60°C. All other parameters were left at their default values.

### 2.3 Plant materials and DNA extraction

The validation materials for polymorphism of SSR markers in this study include four *S.* sp*ontaneum* (Np-X, SES-208, 2013-8, Hainan 2012-124), three *S. officinarum* (Badila, LA-purple, NC20), and three modern sugarcane cultivation materials (XTT22, F134, R570). The validation materials for the universality of SSR markers in this study include nine *S.* sp*ontaneum*, eight *S. officinarum*, 10 modern sugarcane cultivation materials, three *S. robustum*, and three *E. rufipilus.*

Total DNA was extracted from fresh leaves using the plant genomic DNA kit (TIANGEN, China). DNA concentrations were checked using BioPhotometer D30 (Eppendorf, Germany) and the quality of DNA was measured by 1.2% agarose gel electrophoresis.

### 2.4 Polyacrylamide gel electrophoresis (PAGE) run protocol and

Electrophoresis gel preparation: Use 50 mL of non - denaturing polyacrylamide solution for each pair of glass plates. The formula for the 9% solution includes 15 mL of 30% polyacrylamide (29:1), 35 mL of 1×TBE, 500 μL of 10% APS, and 50 μL of TEMED. It takes about 30 minutes to solidify at room temperature. Mix the solutions in a beaker, and pour the gel after the bubbles disappear. After the gel has solidified for about 30 minutes, place the glass plates vertically in the electrophoresis groove, add TBE buffer, and load 2 μL of PCR products with a DNA concentration of 5 ng/μL into the wells in sequence. Load 2 μL of 50 bp Ladder (for PAGE) in the middle well for band comparison. Set the voltage of the electrophoresis apparatus to 150 V and turn off the power after 180 minutes. Prepare the staining solution by adding 10 μL of Gelred to 1000 mL of clean water. After electrophoresis, remove the gel plate, separate the glass plates, make the gel adhere to one plate, immerse it in the staining solution for 10 - 15 minutes, and then observe and take photos using a gel imaging system.

The results of non-denaturing polyacrylamide gel electrophoresis were analyzed. For materials lacking bands at the same amplification site with the same primer pair, a value of 0 was recorded, while materials with bands were recorded as 1. These electrophoresis outcomes were processed to establish a 0 - 1 similarity matrix. Subsequently, the Unweighted Pair - Group Method with Arithmetic Mean (UPGMA) was employed to construct a phylogenetic tree within the NTSYS software.

## 3 Results

### 3.1 Comparison of SSR loci among 14 Poaceae species

In this study, we conducted a search and identification of SSR loci in 14 Poaceae species’ genomes. The genome sizes of these Poaceae species ranged from 271 Mb (*Brachypodium distachyon*) to 8,560 Mb (XTT22, a modern sugarcane cultivar) (**Table 1**). We observed a significant positive correlation between genome size and the number of SSRs (r=0.958). Additionally, there was a significant negative correlation between genome size and the frequency of SSRs (r=-0.461), consistent with previous research findings in flowering plants (Vinatzer et al., 2013).

**Table 1:** Distribution characteristics of SSR loci in 14 Poaceae species.

Species	Genome (Mb)	Number of SSRs	Frequency of SSR/Mb	Type of SSR (%)					
				Mono-	Di-	Tri-	Tetra-	Penta-	Hexa-
Modern sugarcane cultivars (XTT22)	8,560	1,054,918	123	58.86	22.59	9.85	2.61	3.28	2.82
*S. officinarum* (LA-purple)	6,805	806,029	118	55.72	25.27	10.39	2.4	3.41	2.81
*S. officinarum* (Badila)	6,577	800,979	122	58.8	23.22	9.8	2.29	3.22	2.67
*S. spontaneum* (AP85-441)	3,141	387,856	123	59.41	21.81	9.8	2.55	3.34	3.09
*S. spontaneum* (Np-X)	2,761	347,638	126	59.38	21.76	9.9	2.67	3.44	2.85
*E. rufipilus* (YN2009-3)	860	121,436	141	67.38	17.75	8.23	1.86	2.4	2.38
*S. arundinaceum* (HN92-105)	6,728	1,095,633	163	35.01	27.37	9.61	12.06	3.07	12.88
*Hordeum vulgare*	4,341	856,771	197	25.78	17.16	33.96	5.77	1.12	16.2
*Aegilops tauschii*	4,310	522,275	121	36.7	28.97	10.49	11.26	2.17	10.4
*Zea mays*	2,183	233,115	107	34.43	25.08	14.34	9.91	4.49	11.75
*Sorghum bicolor*	709	139,888	197	39.6	22.89	10.24	12.99	3.38	10.89
*Setaria italica*	406	66,678	164	50.4	18.78	10.48	9.02	3.93	7.39
*Oryza sativa*	399	152,466	382	44.46	23.26	9.62	10.75	2.57	9.35
*Brachypodium distachyon*	271	54,144	200	56.65	18.28	10.26	6.51	3.48	4.83

Among the 14 Poaceae plants, rice exhibited the highest SSR frequency (384/Mb), significantly surpassing the average. *Brachypodium distachyon* followed with a frequency of 200/Mb, while *S. officinarum, S. spontaneum*, and cultivated species demonstrated the lowest SSR frequencies (118-126/Mb).

After analyzing the SSR loci characteristics of various Poaceae species, it was discovered that there is some similarity in the distribution of SSR loci among these plants. It was observed that mononucleotide repeats repeat types are the most common SSR types in most Poaceae species (**Table 1**), which is in contrast to previous findings on angiosperms where the most abundant SSR types are three and four nucleotide repeat types (Vinatzer et al., 2013). It is possible that the difference observed is due to different search criteria used when identifying SSRs. For instance, this study considered SSRs with 10 and 11 repeats of mononucleotide repeat type, which was not done in previous studies on angiosperms.

### 3.2 Characteristics of SSR loci in the genome of modern sugarcane cultivars

**Figure 1:**
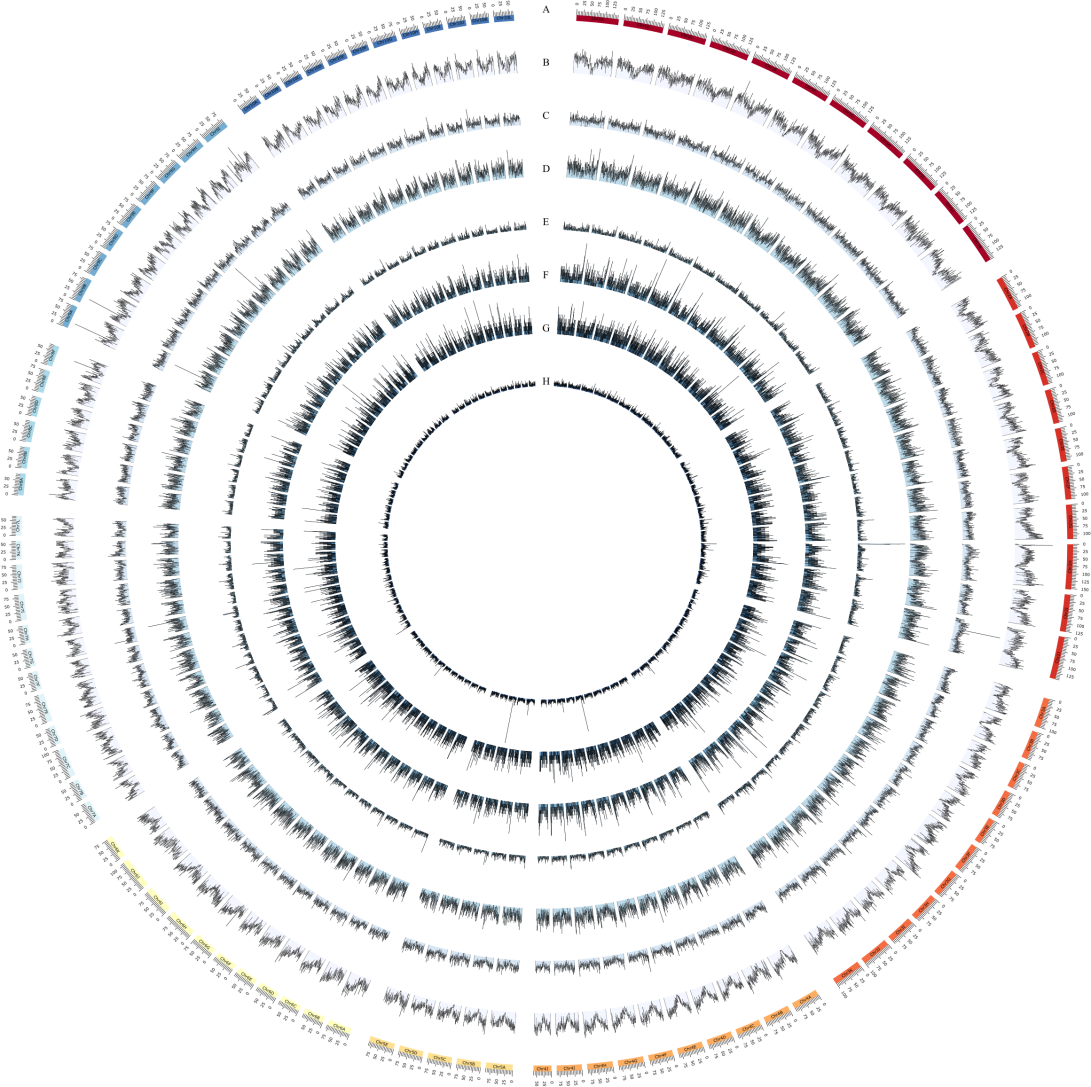
Distribution and frequency of whole genome SSR. **(A)** Chromosome of XTT22. **(B–H)** denote the distribution of Mononucleotide (The density ranges from 0 to 134 SSRs per 500kb), Dinucleotide (The density ranges from 0 to 81 SSRs per 500kb), Trinucleotide (The density ranges from 0 to 28 SSRs per 500kb), Tetranucleotide (The density ranges from 0 to 34 SSRs per 500kb), Pentanucleotide (The density ranges from 0 to 15 SSRs per 500kb), Hexanucleotide (The density ranges from 0 to 12 SSRs per 500kb), and compound (The density ranges from 0 to 180 SSRs per 500kb) repeat SSRs, respectively.

A total of 1,054,918 SSR loci were identified in the genome of XTT22 (**Table 2**; **Figure 1**). Among them, Chr2H (19,000) holds the highest number of SSRs, while Chr7L (6,441) possesses the lowest (**Supplementary Table 1**). These SSR loci consisted of 3,935 different types of SSR motifs, with a frequency of 123 per Mb, equivalent to an average of 1 SSR locus every 8.11 kb. Mononucleotide repeat types were the most abundant, with 620,901 loci, accounting for 58.86% of the total. Dinucleotide and trinucleotide repeat types followed, with 238,261 and 103,920 loci, respectively, representing 22.59% and 9.85% of the total. The proportions of tetranucleotide, pentanucleotide, and hexanucleotide repeat types were all below 5%, with 27,575, 34,565, and 29,696 loci, respectively, accounting for 2.61%, 3.28%, and 2.82% of the total. The A/T type represents the highest proportion among single - nucleotide repeat types, constituting 84.09% of the total number of single - nucleotide repeat types. Regarding dinucleotide repeat types, there are four in total, with AT/TA being the dominant element, accounting for 57.92% of this repeat type. For tetranucleotide and pentanucleotide repeat elements, there are 60 and 235 types respectively, and for hexanucleotide repeat elements, there are 975 types. The major repeat elements include TGT/ACA, AAAT/GATG, and CGAGC/AAAAG. Among all the SSR loci, the minimum number of repeats was 4, and the maximum number of repeats was 522. SSR loci with repeat numbers ranging from 4 to 20 accounted for 88.97% of the total. The most abundant SSR loci had 10 repeats, with a total of 355,762 loci, representing 35.15% of the total. This is mainly due to the fact that the number of mononucleotides repeat loci with 10 repeats is higher than the total number of SSR loci with repeat types ranging from dinucleotide to hexanucleotide repeats.

**Table 2:** Repeat type, number and proportion of SSR in the genome of modern sugarcane cultivar XTT22.

Repeat number	SSR repeat types						Total	Proportion%
	Mono-	Di-	Tri-	Tetra-	Penta-	Hexa-		
4					22,708	19,772	42,480	4.03
5				16,554	6,603	4,944	28,101	2.66
6			54,529	4,407	2,103	1,646	62,685	5.94
7		57,201	20,102	2,003	856	793	80,955	7.67
8		33,848	8,852	1,094	426	490	44,710	4.24
9		20,167	4,474	800	316	348	26,105	2.47
10	355,762	11,488	2,552	513	201	303	370,819	35.15
11	107,554	7,105	1,590	450	133	283	117,115	11.1
12	49,211	4,885	1,047	316	175	198	55,832	5.29
13	27,042	3,970	791	273	114	173	32,363	3.07
14	17,626	3,316	665	180	114	106	22,007	2.09
15	11,558	2,903	538	172	67	97	15,335	1.45
16	7,911	2,640	488	113	63	84	11,299	1.07
17	5,743	2,429	428	118	74	55	8,847	0.84
18	4,390	2,180	432	105	53	58	7,218	0.68
19	3,847	2,109	475	72	53	47	6,603	0.63
20	3,402	2,046	411	65	80	37	6,041	0.57
>20	26,855	81,974	6,546	340	426	262	116,403	11.03
Total	620,901	238,261	103,920	27,575	34,565	29,696	1,054,918	–
Proportion%	58.86	22.59	9.85	2.61	3.28	2.82	–	100
Number of SSR motifs	4	12	60	235	975	2,649	3,935	
Dominance motifs	A/T	AT/TA	TGT/ACA	AAAT/GATG	CGAGC/AAAAG	TAGTGA/ATCACT		
Proportion of dominant motifs%	84.09	57.92	13.06	10.34	7.56	4.42		

**Figure 2:**
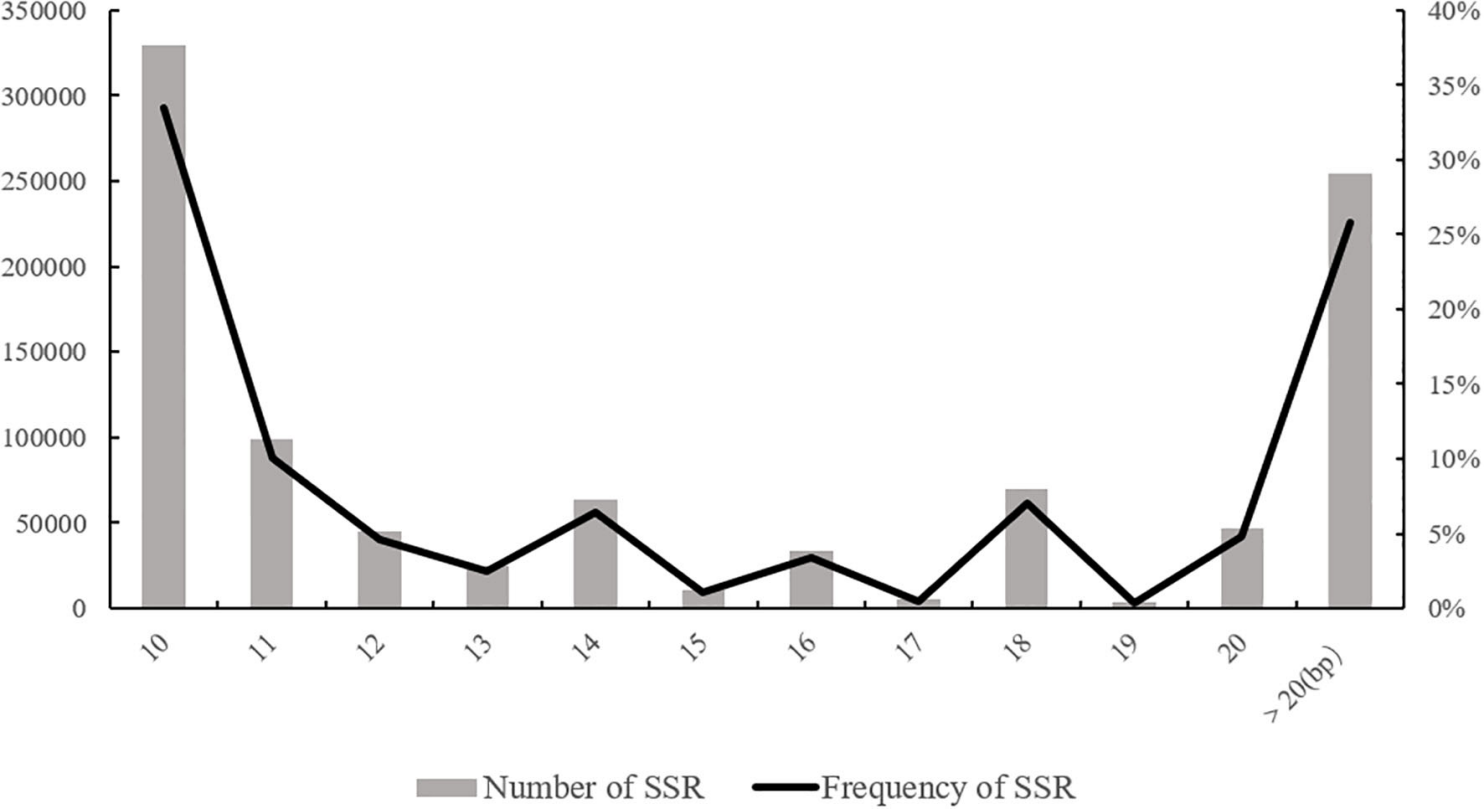
Distribution of SSR motif length in the genome of XTT22.

One of the fundamental factors for the usability of SSR markers is the level of SSR polymorphism, which can be influenced by the length of the SSR motif (Zhang et al., 2022). Criteria for low, moderate, and high levels of polymorphism are typically defined as SSR motif lengths below 12 bp, between 12-20 bp, and above 20 bp, respectively. In this study, the distribution of SSR motif lengths in the genome of the XTT22 was analyzed (**Figure 2**). The highest number of SSR motifs was observed in the range of less than 12 bp (10-11 bp), totaling 329,133 motifs and accounting for 43.51% of the total SSRs. This is mainly due to the abundance of mononucleotide repeat types in this length range. The next most abundant category was motifs with lengths between 12-20 bp, with a count of 301,619 motifs, representing 30.65% of the total. Motifs with lengths exceeding 20 bp had the fewest occurrences, with 254,242 motifs, accounting for 25.84% of the total. These results indicate that the distribution of SSR motif lengths in the entire genome of the modern sugarcane cultivar primarily falls within the range of less than 12 bp, indicating a low level of polymorphism.

Statistical analysis was conducted on the SSR loci in each chromosome of the XTT22. The results showed that Chr01 had the highest number of SSR loci (165,914). The SSR frequency and average distance between two SSRs were statistically analyzed in each chromosome of the XTT22. The results showed that there were 108.07-122.90 SSR loci per Mb, with the highest SSR frequency observed on Chr01 at 122.90 loci/Mb. The average distance between two SSRs on Chr01 was 8.14 kb. On the other hand, Chr05 had the lowest SSR frequency at 102.81 loci/Mb, with an average distance of 9.73 kb between two SSRs.

### 3.3 Development of whole genome level SSR markers in modern sugarcane cultivars

Based on the flanking conserved sequences of SSR loci in the XTT22 genome of modern sugarcane cultivars, a batch design of SSR primers was conducted using Primer3.0 software. A total of 910,519 primer pairs were successfully designed, with 882,677 primer pairs distributed widely distributed on 10 chromosomes. The remaining primer pairs that were not located on chromosomes were not included in the statistical analysis. The frequency of designed primers was 107.43 per Mb, with an average of one SSR primer motif every 9.31 kb. Chr01 had the highest number of SSR primers (154,393), while Chr05 had the fewest (35,502). Among the chromosome, chr03 had the highest SSR frequency, with 114.69 primers per Mb and an average distance of 8.72 kb between two SSRs. Chr05 had the lowest SSR frequency, with 94.42 primers per Mb and an average distance of 10.59 kb between two SSRs.

Subsequently, the amplification efficiency of the 882,677 SSR primer pairs in the XTT22 genome was analyzed using e-PCR. A total of 47,165 primer pairs were able to generate a single amplification product, and these primers were referred to as specific SSR candidate markers. Among them, 35,215 (74.66%) SSR candidate markers were of the standard type, while 11,950 (25.34%) were of the compound type (**Table 3**). Among the specific SSR candidate markers of the standard type, the mononucleotide repeat type accounted for the highest proportion (49.22%), while the four-nucleotide repeat type was the lowest (2.20%). The distribution of specific SSR candidate markers in the XTT22 genome was analyzed, and Chr10 had the highest number of specific SSR candidate markers, with 10,716 primer pairs. This accounted for approximately 50% of all specific SSR candidate markers, indicating that SSR primers had a much better specificity on Chr10 compared to other chromosomes. Chr01 and chr02 followed with 4,469 and 4,293 specific SSR candidate markers, respectively. Chr05 and chr08 had the fewest number of specific SSR candidate markers among all chromosomes, with 373 and 326 specific SSR candidate markers, respectively. This closely correlated with the size of their respective chromosomes (**Table 3**).

**Table 3:** Distribution characteristics of genome-wide specific SSR candidate markers in XTT22.

Chromosome	Mono-	Di-	Tri-	Tetra-	Penta-	Hexa-	Compound	Total
Chr01	2130	481	366	93	133	143	1123	4469
Chr02	2183	404	325	110	147	130	994	4293
Chr03	1478	345	252	65	105	81	759	3085
Chr04	1293	345	241	45	89	87	636	2736
Chr05	662	171	85	33	59	47	373	1430
Chr06	1278	329	219	59	87	87	691	2750
Chr07	1361	308	219	51	99	70	711	2819
Chr08	585	156	122	26	47	49	326	1311
Chr09	1195	330	223	78	99	104	721	2750
Chr10	10716	1864	1170	468	693	482	5454	20847
Total	22881	4733	3222	1028	1558	1280	11788	46490

Among all chromosomes, Chr10 had the highest number of specific SSR candidate marker types. The two most abundant types of specific SSR candidate markers on each chromosome were the mononucleotide e repeat type and the compound type. Their combined quantity exceeded half of the specific SSR candidate markers on each chromosome. However, studies have shown that the mononucleotide repeat type is not suitable for marker development (Cavagnaro et al., 2010). On the other hand, the compound type presents significant challenges and limitations in marker development due to its complex motifs and the possibility of different combinations of repeat numbers within the motifs.

### 3.4 Polymorphism verification of whole genome level SSR markers in modern sugarcane cultivars

**Figure 3:**
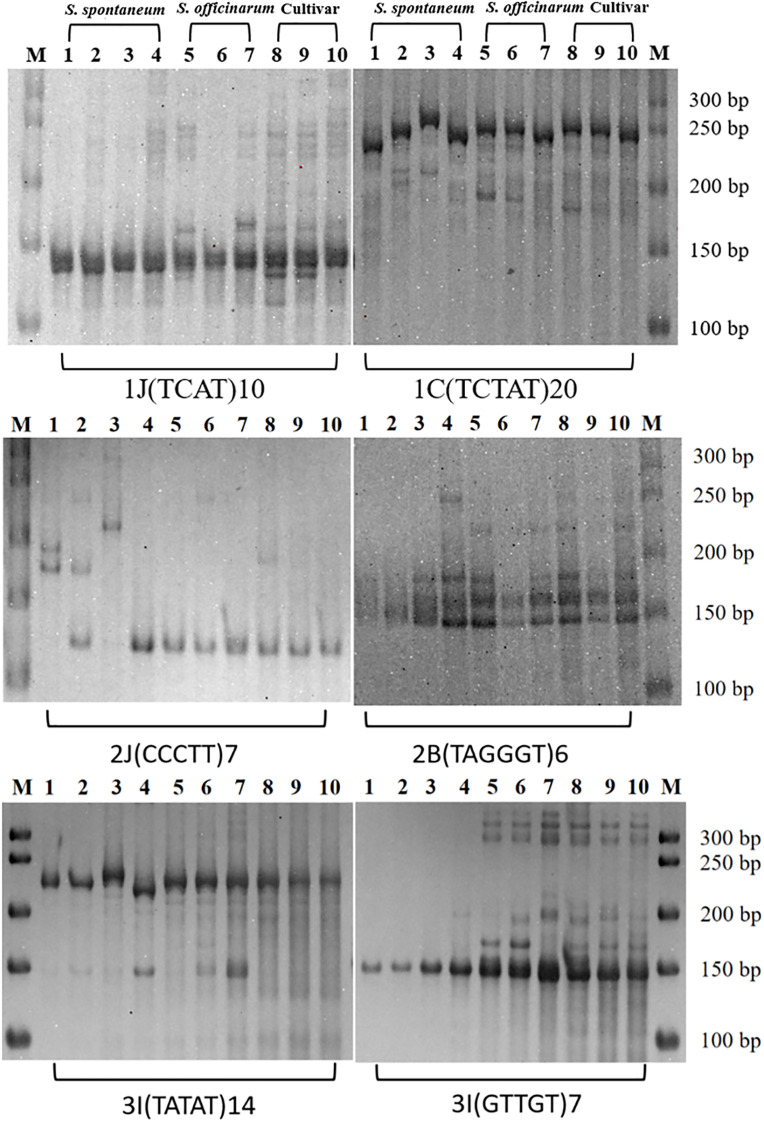
Native-PAGE results of primers 1J(TCAT)10, 1C(TCTAT)20, 2J(CCCTT)7, 2B(TAGGGT)6, 3I(TATAT)14 and 3I(GTTGT)7. 1-10 sugarcane materials were: 1: Np-X; 2: SES208; 3: 2013-8; 4: HN2012-124; 5: Badila; 6: LA purple; 7: NC20; 8: XTT22; 9: F134; 10: R570. M: 50 bp Ladder (for PAGE).

SSR primers with known positions in the genome and specific amplification products have higher utility value. The polymorphism of SSRs is closely related to the length of their motifs. In this study, standard-type specific SSR candidate markers with motif lengths greater than 30 bp were selected from the pool of 47,165 candidates for polymorphism validation in 10 sugarcane materials. A total of 538 specific SSR candidate markers met the screening criteria, and PCR amplification was performed on the 10 different sugarcane materials. The results showed that 507 primer pairs (94.24%) produced amplification products, and among them, 459 primer pairs (85.32%) exhibited polymorphism across the 10 materials (**Figure 3**). These SSR markers had clear and stable bands, making them easy to identify.

The polymorphism rates of the 459 polymorphic markers on each chromosome of the XTT22 genome varied and ranged from 66.67% to 100%. Among them, the highest number of specific SSR candidate markers were screened on chr10 (209 primer pairs), and 182 pairs (87.08%) exhibited polymorphism across the 10 different sugarcane materials. Chr05 and chr08 had the highest polymorphism rates, both reaching 100%. This may be attributed to the relatively smaller number of SSR primers initially developed for these chromosomes. Chr02 and chr06 had relatively lower polymorphism rates, at 66.67% and 76.32%, respectively. The polymorphism rates of SSR markers on other chromosomes showed little difference and were all above 80%. This result indicates that the polymorphic SSR markers developed in this study also exhibit high polymorphism rates at the chromosome level.

A statistical analysis of the different types of SSR repeats among the 459 markers revealed that the pentanucleotide repeat type had the highest number of SSR markers, with 198 primer pairs validated, of which 171 (86.36%) exhibited polymorphism. The number of tetranucleotide and hexanucleotide repeat types was very similar, with 150 and 138 markers, respectively, but they showed significant differences in polymorphism rates (81.97% and 97.90%, respectively). Among the 459 polymorphic SSR markers, there was a diverse range of motif lengths, with the majority falling within the 30-40 bp range. The shortest motif length observed was 32 bp, while the longest reached 192 bp. The highest number of SSR markers was found in the 30-40 bp motif length range, totaling 197 markers. The next highest number of SSR markers was in the 41-50 bp motif length range, with 69 markers. In contrast, the fewest SSR markers were observed in the 81-90 bp motif length range, comprising only 12 markers. When the motif length ranged from 91 to 100 bp, SSR markers exhibited the highest polymorphic rate (90.70%). Furthermore, the polymorphic rates of SSR markers in different motif length ranges exceeded 80%. These results indicate that SSR markers with motif lengths greater than 30 bp hold significant potential for future applications in genetic diversity analysis and kinship identification of different sugarcane varieties.

In this study, experiments were also conducted using 21 core SSR markers developed by the International Society of Sugarcane Technologists (ISSCT) on the 10 sugarcane materials (**Supplementary Table 2**). The results showed that 8 primers amplified clear and distinguishable bands, while 7 primers did not yield any bands across the 10 materials. The remaining markers either showed non-polymorphic bands or weak and unreadable bands. The polymorphism rate of these 21 universal markers across the 10 sugarcane materials was 38.10%, significantly lower than the polymorphic SSR markers developed in this study (85.32%).

### 3.5 Universality analysis of whole genome level SSR markers in modern sugarcane cultivars

*S. officinarum*, characterized by high sugar content but relatively weak stress resistance, and *S.* sp*ontaneum*, known for its strong stress tolerance but lower sugar content in stalks, are the two main ancestral species of modern cultivated sugarcane varieties. Modern cultivated sugarcane varieties combine the strong stress tolerance of *S.* sp*ontaneum* with the high sugar content of *S. officinarum*. The polymorphism of SSR markers has been validated in materials of S. officinarum, *S.* sp*ontaneum*, and cultivated sugarcane varieties. To investigate whether these SSR markers are applicable in a wider range of sugarcane species and their related genera, 24 SSR markers were randomly selected for testing their universality in materials of *S.* sp*ontaneum*, *S. officinarum*, cultivated sugarcane, *S. robustum*, and other closely related species within the genus Saccharum.

The sequence information, number of amplified alleles (Na), and polymorphic information content (PIC) of the 24 polymorphic SSR markers were statistically analyzed (**Supplementary Table 3**). These markers amplified 134 different alleles across 33 sugarcane and related species materials, with an average of 5.58 alleles per primer. The PIC value reflects the ability of SSR markers to differentiate populations. If PIC is greater than 0.5, the SSR marker is considered highly polymorphic. Within the range of 0.25-0.5, it is considered moderately polymorphic. If PIC is less than 0.25, it is considered low polymorphic. The developed 24 polymorphic SSR markers in this study exhibited a PIC range of 0.45-0.95, with an average PIC value of 0.75, indicating rich polymorphism in the amplified bands. None of the SSR markers had a PIC value below 0.25, while two markers had PIC values between 0.25 and 0.5. The remaining 22 markers had PIC values exceeding 0.5, indicating highly polymorphic markers. This suggests that these SSR markers developed of this study can play a crucial role in molecular breeding of sugarcane.

**Figure 4:**
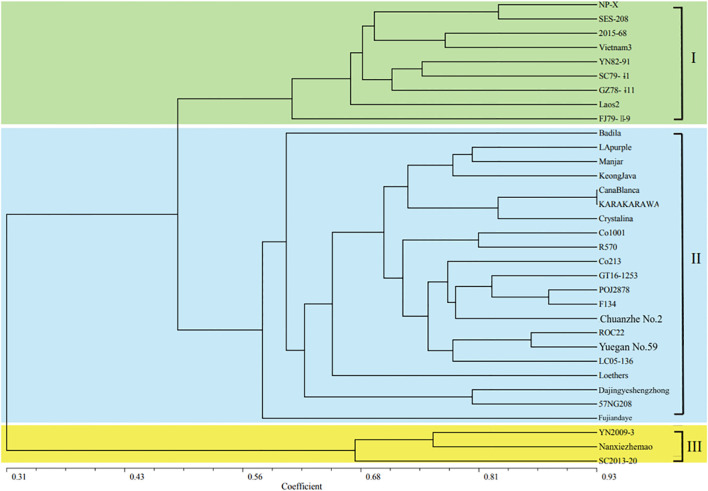
Cluster analysis of thirty *Saccharum* and three *Erianthus* materials.

The cluster diagram (**Figure 4**) of the 33 sugarcane materials were calculated using the NTSYSPC2.1 software. The results showed that the genetic similarity coefficients ranged from 0.19 to 0.93, with an average of 0.58, indicating significant genetic variation among these sugarcane and related species materials. *S. officinarum* (Keong Java) and the *E. rufipilus* (YN2009-3 exhibited) the lowest genetic similarity coefficient of 0.19, indicating a large genetic distance and distant relationship between these two materials. On the other hand, the two *S. officinarum* KARA KARA WA and Cana Blanca had the highest genetic similarity coefficient of 0.93, indicating a close genetic distance and highly similar genetic backgrounds between these two materials. The 24 SSR markers successfully differentiated the 33 different sugarcane materials (**Figure 4**). When the genetic similarity coefficient reached 0.56, these materials could be classified into three main groups: the first group consisted of 9 *S.* sp*ontaneum*, the second group consisted of 21 materials including 8 *S. officinarum*, 10 cultivated varieties, and 3 *S. robustum*, and the third group consisted of 3 *E. rufipilus* materials. This indicates that the SSR markers developed in this study can be used for genetic relationship identification, cultivar identification, and genetic diversity analysis among sugarcane and its related species. Molecular markers can provide insights into the genetic background of sugarcane varieties with unclear pedigrees or unknown origins, enabling more targeted parental selection and guidance for breeding purposes.

## 4 Discussion

### 4.1 Evaluation of SSR density and characterization in whole-genome of modern cultivated sugarcane

Among the 14 Poaceae species analyzed, excluding *Hordeum vulgare*, the most abundant repeat type was the mononucleotide repeat type, followed by the dinucleotide repeat type. This finding is consistent with the results reported for *Diospyros oleifera* (Xu et al., 2023), strawberry (Miao et al., 2021), and *Bunium persicum* (Bansal et al., 2022), but differs with cotton (Zou et al., 2012). *S. officinarum*, *S.* sp*ontaneum*, and modern cultivated sugarcane showed similar patterns in terms of nucleotide repeat type content, except for the tetranucleotide repeat type, which showed some variations. In most Poaceae species, the most abundant repeat motif in the mononucleotide repeat type was A/T, except for *Hordeum vulgare*, *Aegilops tauschii*, and *Zea mays*, where G/C content was highest. In the dinucleotide repeat type, most Poaceae species had AT/TA as the most abundant motif, which is similar to soybean (Blair et al., 2008), while foxtail millet and *Brachypodium distachyon* had GG/CC as the most frequent motif. In the trinucleotide repeat type, TGT/ACA was the most common motif *S. officinarum*, *S.* sp*ontaneum*, and modern cultivated sugarcane, which is different from loquat (Jiang et al., 2021), where AAG/CTT had the highest proportion. Overall, in most of the analyzed Poaceae species, the most common repeat types in the mononucleotide to trinucleotide repeat motifs contained A and T nucleotides, which is consistent with the results observed in eukaryotes. This could be attributed to the fact that during DNA replication, the hydrogen bonds between A and T require less energy compared to those between G and C, making the A/T structure of SSR motifs more prone to occur.

Microsatellite is an important mutation in the genome because of its wide distribution and high frequency, which plays a significant role in genomics research (Tribhuvan et al., 2019). The wide distribution and high frequency of microsatellites in the genome indicate their universality and importance. The high frequency of these repetitive sequences suggests their potential role in genetic variation and evolutionary processes (Zhao et al., 2023). These findings contribute to our understanding of the genomic features of microsatellites in plants and their relevance in genomic research (Xu et al., 2021b). Prior research has determined that SSR significantly contributes to genome plasticity and environmental adaptation (Yuan et al., 2021). Given the high complexity of modern sugarcane cultivars’ genomes, the identification and utilization of SSR loci could potentially establish a foundation for unraveling the genomic evolution of these modern sugarcane cultivars.

### 4.2 Potential application of SSR markers of molecular breeding in sugarcane

SSR markers are often used in genetic research and molecular marker-assisted (MAS) breeding. Among the 538 pairs of specific SSR primers developed in this study, a total of 507 primer pairs showed amplification products, resulting in an amplification efficiency of 94.24%. This efficiency was higher than the SSR primer amplification efficiency reported by Wang et al. (2020) based on the haploid cultivar R570 (90.24%) and Gordeiro et al. (2001) based on the sugarcane EST library (60.00%). Additionally, out of the 459 primer pairs, 459 showed polymorphisms in 10 sugarcane materials, with a polymorphic rate of 85.32% (459/538). This polymorphic rate was significantly higher than the polymorphic rate of 32.62% (349/1070) reported by Xiao et al. (2020) for EST-SSR markers in 12 sugarcane materials. The higher polymorphic rate observed in the SSR markers developed from the sugarcane whole-genome sequence in this study compared to the EST-SSR markers developed from the sugarcane EST library by previous researchers may be attributed to the fact that EST-SSR markers mainly represent polymorphisms in gene coding regions (Paliwal et al., 2022), whereas the whole-genome SSR markers are predominantly distributed in non-coding regions, where the mutation rate may be higher than in coding regions. Further, 21 core SSR markers developed by the International Society of Sugar Cane Technologists (ISSCT) were utilized. These markers belong to the first molecular identification database for sugarcane based on SSR markers in the world (Pan, 2010). They were widely used in previous studies when sugarcane genome data were not available. However, with the continuous development of sugarcane genome sequencing, the limited coverage and low representation of these markers in the sugarcane genome have become apparent. Therefore, there is a need for SSR markers with broader coverage and higher polymorphism to serve molecular marker-assisted breeding and genetic mapping in sugarcane. The SSR candidate markers developed in this study are distributed across the chromosomes of modern sugarcane cultivars and exhibit high coverage. They can provide directions for future investigations into the genetic relationships of sugarcane hybrids at the chromosomal level.

*S. officinarum* serve as the source of high sucrose genes in modern cultivated sugarcane, while *S.* sp*ontaneum* provides resistance traits, making it the most valuable wild species in sugarcane breeding (Jackson, 2005). Modern cultivated sugarcane combines the strong stress resistance of *S.* sp*ontaneum* and the high sucrose content of *S. officinarum* (Zhang et al., 2018a). Among the selected modern sugarcane cultivars, POJ2878, F134, Chuanzhe 2, and XTT22 represent three stages of parental replacement in sugarcane breeding in China and are considered backbone parents (Zhang et al., 2009). *S. robustum*, with its advantages of insect resistance, lodging resistance, and drought tolerance, is an important wild species in the Saccharum genus and highly valued by sugarcane breeders (Yu et al., 2023). *E. rufipilus*, a closely related species of sugarcane, has excellent characteristics of drought tolerance, cold tolerance, and disease resistance (Wang et al., 2010) and has been used in sugarcane breeding through interspecific hybridization. The three selected Miscanthus materials are of great value in sugarcane breeding research, with Yunnan 2009-3, a diploid *E. rufipilus*, having available genomic data (Wang et al., 2023b), which will play a significant role in the analysis of sugarcane origin and evolution in the future. The SSR markers selected in this study have important research value in the experimental validation materials. Therefore, the identified universal SSR markers can provide a certain number of markers for breeding research in these materials and serve as a reference for selecting more optimized parents in future sugarcane breeding.

## 5 Conclusion

SSR markers have been widely used in various aspects of sugarcane research, including genetic diversity analysis, variety identification, kinship analysis, and QTL mapping, due to their co-dominance, genome-wide coverage, high polymorphism, and good experimental reproducibility. In this study, SSR loci were identified in the genomes of 14 Poaceae species for the first time. It was found that there was a positive correlation between genome size and the number of SSRs, and a negative correlation between genome size and the frequency of SSRs. Mononucleotide repeats were common in most Poaceae plants. A total of 1,054,918 SSR loci were identified in the genome of the modern sugarcane cultivar XTT22. A total of 910,519 pairs of primers were developed, and after screening, 459 polymorphic SSR markers were obtained. The polymorphism rate of these markers was as high as 85.32%, which was significantly higher than that of the universal markers (38.10%) developed by the International Society of Sugarcane Technologists. The 24 SSR markers showed rich polymorphism in 33 sugarcane and related species materials, and could effectively distinguish these materials. These markers can be used for the identification of genetic relationships, cultivar discrimination, and genetic diversity analysis of sugarcane and related species, providing crucial support for molecular breeding of sugarcane.

## Data Availability

The datasets presented in this study can be found in online repositories. The names of the repository/repositories and accession number(s) can be found in the article/**Supplementary Material**.
