# Supplementary material for: Genome-wide development of SSR molecular markers for modern sugarcane cultivars
Source: Front Plant Sci. 2025 Apr 1;16:1573967. doi: 10.3389/fpls.2025.1573967 (PMC11996920; doi:10.3389/fpls.2025.1573967)
Supplement: Supplementary file 1 [file DataSheet1.docx]

Supplementary Table 1. Distribution characteristics of SSR loci on chromosomes in XTT22 genome

| Chromosome | | Number of SSRs | Size of chromosome/Mb | SSR density/(SSR/Mb) |
| --- | --- | --- | --- | --- |
| Chr1A | 18,238 | | 143.82 | 126.81 |
| Chr1B | 16,634 | | 135.87 | 122.42 |
| Chr1C | 16,803 | | 132.04 | 127.26 |
| Chr1D | 17,636 | | 135.62 | 130.04 |
| Chr1E | 18,072 | | 135.98 | 132.90 |
| Chr1F | 16,246 | | 131.56 | 123.49 |
| Chr1G | 17,514 | | 140.88 | 124.32 |
| Chr1H | 18,199 | | 144.11 | 126.29 |
| Chr1I | 17,449 | | 137.02 | 127.35 |
| Chr1J | 16,827 | | 133.02 | 126.50 |
| Chr1K | 17,421 | | 140.08 | 124.36 |
| Chr2A | 14,068 | | 118.73 | 118.48 |
| Chr2B | 14,601 | | 124.85 | 116.95 |
| Chr2C | 13,854 | | 117.07 | 118.34 |
| Chr2D | 14,030 | | 117.61 | 119.30 |
| Chr2E | 13,699 | | 114.78 | 119.35 |
| Chr2F | 13,569 | | 112.01 | 121.14 |
| Chr2G | 15,422 | | 117.33 | 131.44 |
| Chr2H | 19,000 | | 153.38 | 123.87 |
| Chr2I | 16,034 | | 126.83 | 126.42 |
| Chr2J | 17,496 | | 145.67 | 120.11 |
| Chr3A | 12,721 | | 101.90 | 124.84 |
| Chr3B | 10,837 | | 96.84 | 111.91 |
| Chr3C | 11,471 | | 92.15 | 124.48 |
| Chr3D | 11,684 | | 91.34 | 127.92 |
| Chr3E | 11,074 | | 89.77 | 123.36 |
| Chr3F | 12,473 | | 95.69 | 130.35 |
| Chr3G | 12,556 | | 92.56 | 135.65 |
| Chr3H | 11,499 | | 93.87 | 122.50 |
| Chr3I | 11,870 | | 91.90 | 129.16 |
| Chr3J | 13,491 | | 101.14 | 133.39 |
| Chr3K | 12,166 | | 101.62 | 119.72 |
| Chr4A | 10,996 | | 92.40 | 119.01 |
| Chr4B | 10,432 | | 91.43 | 114.09 |
| Chr4C | 9,685 | | 86.28 | 112.25 |
| Chr4D | 10,343 | | 87.52 | 118.18 |
| Chr4E | 10,577 | | 87.49 | 120.89 |
| Chr4F | 10,381 | | 83.75 | 123.95 |
| Chr4G | 10,261 | | 88.06 | 116.53 |
| Chr4H | 11,372 | | 87.68 | 129.69 |
| Chr4I | 10,349 | | 86.11 | 120.18 |
| Chr4J | 7,480 | | 61.25 | 122.13 |
| Chr5A | 10,294 | | 91.34 | 112.70 |
| Chr5B | 8,914 | | 82.06 | 108.62 |
| Chr5C | 8,769 | | 82.54 | 106.23 |
| Chr5D | 8,574 | | 82.80 | 103.55 |
| Chr5E | 8,497 | | 87.29 | 97.34 |
| Chr6A | 8,972 | | 80.60 | 111.32 |
| Chr6B | 8,185 | | 73.36 | 111.56 |
| Chr6C | 8,077 | | 70.01 | 115.38 |
| Chr6D | 8,161 | | 70.19 | 116.26 |
| Chr6E | 8,434 | | 70.25 | 120.05 |
| Chr6F | 8,182 | | 70.72 | 115.69 |
| Chr6G | 8,829 | | 77.69 | 113.65 |
| Chr6H | 11,850 | | 96.03 | 123.40 |
| Chr6I | 11,586 | | 94.67 | 122.39 |
| Chr6J | 12,527 | | 108.86 | 115.08 |
| Chr6K | 9,690 | | 79.29 | 122.20 |
| Chr7A | 7,838 | | 70.59 | 111.04 |
| Chr7B | 7,145 | | 66.78 | 107.00 |
| Chr7C | 10,837 | | 100.80 | 107.51 |
| Chr7D | 7,501 | | 64.96 | 115.48 |
| Chr7E | 11,048 | | 96.47 | 114.52 |
| Chr7F | 7,381 | | 63.31 | 116.59 |
| Chr7G | 7,431 | | 62.68 | 118.55 |
| Chr7H | 6,951 | | 62.96 | 110.39 |
| Chr7I | 10,292 | | 88.19 | 116.71 |
| Chr7J | 9,729 | | 83.19 | 116.95 |
| Chr7K | 7,050 | | 63.34 | 111.31 |
| Chr7L | 6,441 | | 66.38 | 97.03 |
| Chr8A | 8,639 | | 74.57 | 115.85 |
| Chr8B | 8,197 | | 73.61 | 111.36 |
| Chr8C | 8,351 | | 71.37 | 117.01 |
| Chr8D | 7,847 | | 67.23 | 116.72 |
| Chr8E | 9,069 | | 77.58 | 116.90 |
| Chr8F | 7,380 | | 71.59 | 103.08 |
| Chr9A | 8,667 | | 78.57 | 110.31 |
| Chr9B | 9,408 | | 84.97 | 110.72 |
| Chr9C | 9,532 | | 82.41 | 115.68 |
| Chr9D | 9,105 | | 79.27 | 114.86 |
| Chr9E | 9,867 | | 83.45 | 118.23 |
| Chr9F | 8,532 | | 80.63 | 105.82 |
| Chr9G | 9,652 | | 82.99 | 116.31 |
| Chr9H | 9,905 | | 86.94 | 113.94 |
| Chr9I | 10,235 | | 87.89 | 116.44 |
| Chr10A | 8,310 | | 69.03 | 120.38 |
| Chr10B | 7,478 | | 64.82 | 115.37 |
| Chr10C | 7,404 | | 64.98 | 113.94 |
| Chr10D | 7,944 | | 65.30 | 121.66 |
| Chr10E | 8,347 | | 68.17 | 122.45 |
| Chr10F | 7,482 | | 61.43 | 121.81 |
| Chr10G | 9,806 | | 82.72 | 118.56 |
| Chr10H | 7,775 | | 63.41 | 122.61 |
| Chr10I | 7,840 | | 63.24 | 123.97 |
| Chr10J | 7,652 | | 62.18 | 123.06 |
| Chr10K | 6,941 | | 60.69 | 114.37 |
| Chr10L | 7,837 | | 68.12 | 115.04 |

Supplementary Table 2. Primer pairs of 21 highly polymorphic SSR markers developed by the International Consortium of Sugarcane Biotechnologists

| Number | SSR marker | Primer sequence（5'-3'） | Motif | Tm（℃） | Size（bp） | Motif length（bp） |
| --- | --- | --- | --- | --- | --- | --- |
| 1 | SMC18SA | ATTCGGCTCGACCTCGGGAT/AGTCGAAAGGTATAATAGTGTTAC | (CGA)10 | 62 | 137-150 | 30 |
| 2 | SMC336BS | ATTCTAGTGCCAATCCATCTCA/CATGCCAACTTCCAAACAGAC | (TG)23(AG)19 | 62 | 141-183 | 84 |
| 3 | SMC22DUQ | CCATTCGACGAAAGCGTCCT/CAAGCGTTGTGCTGCCGAGT | (CAG)5C(AGG)5 | 62 | 148-163 | 31 |
| 4 | SMC278CS | TTCTAGTGCCAATCCATCTCAGA/CATGCCAACTTCCAAACAGACT | (TG)19(AG)25 | 64 | 140-182 | 88 |
| 5 | SMC569CS | GCGATGGTTCCTATGCAACTT/TTCGTGGCTGAGATTCACACTA | (TG)37 | 62 | 167-222 | 74 |
| 6 | SMC1604SA | AGGGAAAAGGTAGCCTTGG/TTCCAACAGACTTGGGTGG | (TGC)7 | 58 | 109-124 | 21 |
| 7 | mSSCIR3 | ATAGCTCCCACACCAAATGC/GGACTACTCCACAATGATGC | (GT)28 | 60 | 141-187 | 56 |
| 8 | SMC334BS | CAATTCTGACCGTGCAAAGAT/CGATGAGCTTGATTGCGAATG | (TG)36 | 60 | 146-164 | 72 |
| 9 | SMC1751CL | GCCATGCCCATGCTAAAGAT/ACGTTGGTCCCGGAACCG | (TGC)7 | 60 | 140-151 | 21 |
| 10 | mSSCIR74 | GCGCAAGCCACACTGAGA/ACGCAACGCAAAACAACG | (CGC)9 | 54 | 217-229 | 27 |
| 11 | mSSCIR43 | ATTCAACGATTTTCACGAG/AACCTAGCAATTTACAAGAG | (GT)3(AT)2(GT)29 | 52 | 206-252 | 68 |
| 12 | mSSCIR66 | AGGTGATTTAGCAGCATA/CACAAATAAACCCAATGA | (GT)43GC(GT)6 | 48 | 127-134 | 100 |
| 13 | SMC31CUQ | CATGCCAACTTCCAATACAGACT/AGTGCCAATCCATCTCAGAGA | (TC)10(AC)22 | 62 | 138-179 | 64 |
| 14 | SMC703BS | GCCTTTCTCCAAACCAATTAGT/GTTGTTTATGGAATGGTGAGGA | (CA)12 | 62 | 206-222 | 24 |
| 15 | SMC851MS | ACTAAAATGGCAAGGGTGGT/CGTGAGCCCACATATCATGC | (AG)29 | 58 | 128-141 | 58 |
| 16 | SMC7CUQ | GCCAAAGCAAGGGTCACTAGA/AGCTCTATCAGTTGAAACCGA | (CA)10(C)4 | 60 | 158-170 | 24 |
| 17 | SMC36BUQ | GGGTTTCATCTCTAGCCTACC/TCAGTAGCAGAGTCAGACGCTT | (TTG)7 | 64 | 112-121 | 21 |
| 18 | SMC597CS | GCACACCACTCGAATAACGGAT/AGTATATCGTCCCTGGCATTCA | (AG)31 | 64 | 144-168 | 62 |
| 19 | SMC24DUQ | CGCAACGACATATACACTTCGG/CGACATCACGGAGCAATCAGT | (TG)13 | 64 | 126-142 | 26 |
| 20 | SMC119CG | TTCATCTCTAGCCTACCCCAA/AGCAGCCATTTACCCAGGA | (TTG)12 | 58 | 106-131 | 36 |
| 21 | SMC486CG | GAAATTGCCTCCCAGGATTA/CCAACTTGAGAATTGAGATTCG | (CA)34 | 58 | 224-241 | 68 |

Supplementary Table 3. Information of 24 polymorphic SSR markers

| Number | Primer | SSR motif | Left primer sequence（5’-3’) | Right primer sequence（5’-3’) | Na | PIC |
| --- | --- | --- | --- | --- | --- | --- |
| 1 | 1A(ATAGA)20 | ATAGA | TGTCACAATCCTTGTTCGGA | TCGGCAAGCACACTATTGAC | 7 | 0.73 |
| 2 | 1B(TATT)8 | TATT | TCGCACACCAACCATAAGAG | TCCTTATCCGACATTGCACA | 8 | 0.85 |
| 3 | 2A(GGAGGT)6 | GGAGGT | TCACGTGAAGGACAGACTGG | CAGTCCTTCTAGTACCGCCG | 2 | 0.5 |
| 4 | 2J(CCCTT)7 | CCCTT | TCCCATGTGCTTTTTCACTG | ATGGAGGAAAGGGAGGGAG | 5 | 0.52 |
| 5 | 3F(AAAT)9 | AAAT | GCGACTCACGTCTATGGTTG | GCCAACGATTAATGCAACTG | 4 | 0.72 |
| 6 | 3I(GTTGG)7 | GTTGG | CCTTGCAGGCACTAGAGGTC | CACCAGGCTCACACAAACAC | 5 | 0.69 |
| 7 | 3I(TATT)11 | TATT | TGTTCGCTGTCATCACTTTGA | ATTTCCTCCTCCATCGGATT | 1 | 0.71 |
| 8 | 4A(AAAT)11 | AAAT | CAACTAAACGGCACACATGG | GGATGATGATCTGGTTGGCT | 4 | 0.74 |
| 9 | 4E(TGGGAC)6 | TGGGAC | GGACACAAGTGGAGCACGTA | CCCAATCCCACATCTGAATC | 6 | 0.75 |
| 10 | 5B(TATAT)7 | TATAT | TGTCTGCTTCTCCAATGCAA | CCTCCAGACTTCACTCCAGC | 7 | 0.86 |
| 11 | 5B(TGAGA)7 | TGAGA | TTCTACCTTCCCCCAGGTTT | CTCATCCACACATTCACCCA | 7 | 0.74 |
| 12 | 5D(TGAATA)6 | TGAATA | CAGCTAACTGTCGGAGTCCC | AACCCGAATTCATTCATCCA | 11 | 0.89 |
| 13 | 6A(TAATA)14 | TAATA | GGAAGCCCTAGCCTTGAAAC | GGCTAAGGTCCAATCCCATT | 2 | 0.95 |
| 14 | 6K(ATGGAG)9 | ATGGAG | AGCAACAACAATGGAGGAGG | GCAAAAGTAGGCAACTAGCCA | 1 | 0.45 |
| 15 | 7A(TCTA)38 | TCTA | CGTTTCATATTGTCGGTACTTG | CGACACGGAGTAACACATATCC | 5 | 0.8 |
| 16 | 7E(ATAGTA)10 | ATAGTA | GCACGGGCTGTGTAGGTTAT | GCGCGGAATCTAGGAGCTAT | 5 | 0.65 |
| 17 | 7L(AATA)9 | AATA | CGAAATGCAGTAACAAAGTTGG | GAGGACGTCTTTTCCAGCAG | 3 | 0.59 |
| 18 | 8B(CATCTC)8 | CATCTC | GCAAAAGTAGGCAACTAGCCA | AGCAACAACAATGGAGGAGG | 7 | 0.86 |
| 19 | 8D(CAAAA)7 | CAAAA | GAACAAGCCATCAACATCCC | CAAGTTCAGAGTGCTCGTGG | 7 | 0.84 |
| 20 | 9A(ATACAT)12 | ATACAT | TGCCCTCAACCTTTACATCC | GACGGTATGGCATGTGTCTG | 10 | 0.87 |
| 21 | 9B(CTTT)11 | CTTT | AGCCAGTTTCATTATTGGGC | GAGGGAATGGTTGTTGCATT | 6 | 0.83 |
| 22 | 9F(AATAT)15 | AATAT | ATGCATGTGTCTTCTCGCAG | AAAGTGCCTTGCTTCGTCAT | 10 | 0.86 |
| 23 | 10B(ATAA)14 | ATAA | AGCCTGCTCAGCCAGATAAG | TGGGAGGGAAAGTAAAACCA | 6 | 0.81 |
| 24 | 10D(AGAT)10 | AGAT | ATTTTGGGCTCTGTTCCTGA | TAATGTAAGTGGGCCTGGGA | 5 | 0.72 |
